# A decade of experience with injuries to the gallbladder

**DOI:** 10.1186/1752-2897-4-3

**Published:** 2010-04-15

**Authors:** Chad G Ball, Elijah Dixon, Andrew W Kirkpatrick, Francis R Sutherland, Kevin B Laupland, David V Feliciano

**Affiliations:** 1 Department of Surgery, University of Calgary, Calgary, Alberta, Canada; 2 Department of Critical Care Medicine, University of Calgary, Calgary, Alberta, Canada; 3 Department of Surgery, Emory University, Atlanta, Georgia, USA

## Abstract

**Background:**

Considering that injuries to the gallbladder are rare, the purpose of this study was to evaluate injury patterns, operative procedures and outcomes in patients with trauma to the gallbladder. A retrospective review of traumatic injuries to the gallbladder at an urban level 1 trauma center from 1996 to 2008 was performed. Injuries were identified via imaging or during operative exploration.

**Results:**

Injuries to the gallbladder occurred in 45 patients, 40 (89%) of whom suffered penetrating trauma. Associated injuries were present in 44 (98%) patients, including 10 (22%) pancreatic injuries requiring repair and/or drainage. Patients were severely injured (49% hemodynamically unstable at presentation; mean Injury Severity Score = 20; mean length of stay = 22 days; mortality rate = 24%). Cholecystectomy was performed in 42 patients (93%), while the remaining 3 had drainage only as part of a "damage control" operation related to their critical physiologic status. Injuries to the extrahepatic biliary ducts occurred in 3 patients (7%) as well. Although all patients developed trauma related complications, none were a direct result of their biliary tract injuries.

**Conclusion:**

Injuries to the gallbladder are rare even in the busiest urban trauma centers. Almost all patients have associated intra-abdominal injuries, and nearly 50% of patients are hemodynamically unstable on admission. Rapid cholecystectomy is the treatment of choice for all mechanisms of injury, except when the first operative procedure is of the damage control type.

## Introduction

Trauma to the gallbladder is rare even in the busiest centers. This likely relates to its inherent surrounding protection afforded by the liver, intestines, omentum and thoracic cage. As a result, injuries tend to be a result of direct blows, acceleration/deceleration shearing, or more commonly penetrating mechanisms [1,2]. Gallbladder trauma also tends to occur in association with multiple concurrent intra-abdominal injuries [1,2].

Although the gallbladder plays a central role in the storage, concentration and regulation of bile, it is also an organ that can be removed [3]. Furthermore, because concurrent gallbladder diseases such as cholecystitis or choleduodenal fistulas are extremely rare in the injured patient, cholecystectomies can be completed rapidly. This procedure is extremely safe, with an associated bile duct injury rate of less than 1% [2] and the occurrence of associated chronic post-cholecystectomy diarrhea in 5% to 18% of patients [4,5].

The primary goal of this study was to evaluate injury patterns, operative procedures and outcomes in patients with trauma to the gallbladder.

## Methods

The study population consisted of all patients (blunt and penetrating) treated at level 1 urban trauma center (Grady Memorial Hospital), between January 1, 1996 and December 31, 2008, with injuries to their gallbladder. Patient demographics, injury characteristics and outcomes were each obtained via the trauma registry and patient charts. Exclusion criteria included any patient without a gallbladder injury, as well as those who arrived without signs of life. This retrospective analysis was intended to be primarily descriptive. It was also approved by our institutional ethics review board.

Analysis was performed using Stata version 8.0 (Stata Corp, College Station, TX.). Normally or near-normally distributed variables were reported as means and non-normally distributed variables as medians. Means were compared using the student's t test and medians using the Mann-Whitney U test. Differences in proportions among categorical data were assessed using Fischer's exact test. A p value less than 0.05 was considered to represent statistical significance for all comparisons.

## Results

Records for 45 patients with gallbladder injuries were available for the 13-year study period (Table 1). Penetrating mechanisms were responsible for 89% of cases (24 gunshot wounds, 16 stab wounds). The remaining 5 patients were struck by vehicles (3) or involved in motor vehicle crashes (2). The prevalence of gallbladder injuries remained consistent across the study interval.

**Table 1:** Patient demographics and injury characteristics

Total patients	45
Mean Age	27 years (SD: 8.69)
Male	87%
Median Injury Severity Score	20 (range: 9-41)
Hemodynamic Instability	49%
Median Length of Stay (hospital)	22 days (range: 0-108)
Median Length of Stay (ICU)	12 days (range: 1-107)
Mortality	24%
Penetrating Mechanism of Injury	89%
Gunshot Wounds	24
Stab Wounds	16
Pedestrians Struck	3
Motor Vehicle Collisions	2
Associated Intraabdominal Injuries	
Liver	38
Large Bowel	18
Kidney	12
Pancreas	10
Small Bowel	8
Stomach	7
Abdominal Vascular	5
Extrahepatic Biliary Tree	3
Spleen	1
Associated Non-Intraabdominal Injuries	
Extremity Non-Vascular	11
Extremity Vascular	5
Traumatic Brain Injuries	4
Pulmonary	3
Extremity Fractures	2
Cardiac	1

Patients with gallbladder injuries were 87% (39/45) male with a mean age of 27 years (SD: 8.69). The median injury severity score (ISS) was 20 (range: 9-41), with an associated median length of hospital stay of 22 days (range: 0-108). The median length of stay in the ICU was 12 days (range: 1-107). Many patients (49%) presented to the hospital with hemodynamic instability. These patients were transported to the operating room immediately. Gallbladder injuries were diagnosed preoperatively in 7 (16%) patients (CT imaging), and incidentally during operative exploration in the remaining 38 patients. The Focused Assessment with Sonography for Trauma (FAST) examination was positive in 88% (7/8) of patients with a preoperative CT diagnosis of a gallbladder injury. Overall mortality was 24% (11/45). Of the 11 patients who died, causes included exsanguinating hemorrhage secondary to either abdominal vascular (3) or thoracic/cardiac (3) injuries, delayed complications of multi-organ failure (3), and traumatic brain injuries (2). Associated injuries were present in 44 (98%) patients, including 10 (22%) pancreatic injuries (Table 1). Of these patients, 5 underwent pancreatic resections and 5 received peri-pancreatic drainage only. Three different patients (7%) also had injuries to their extrahepatic biliary ducts (2 common bile ducts and 1 common hepatic duct). The surviving patient underwent a choledocojejunostomy reconstruction. Remaining operative procedures included repair and/or excision of the above mentioned injuries (partial hepatectomy/resectional hepatic debridement (12), colon repair and/or partial colectomy (18), nephrectomy (8), renal repair (3), small bowel repair and/or excision (8), gastric repair (4), partial gastrectomy (3), abdominal vascular repair (5), and a splenectomy (1)). Nine patients required a repeat laparotomy. Non-abdominal procedures included extremity vascular repair (4), fasciotomy (4), amputation (1), intramedullary nailing (2), thoracotomy with pulmonary resection (2), sternotomy with cardiac repair and pulmonary wedge resection (1), and a craniotomy (1).

Cholecystectomy was performed in 42 (93%) patients. The remaining 3 underwent local drainage only as part of an initial damage control procedure in patients displaying physiologic exhaustion secondary to concurrent abdominal vascular hemorrhage. Both survivors received a cholecystectomy during the second operative procedure. Outside of the 2 damage control survivors, all cholecystectomies were performed immediately during the first operative procedure (<6 hours after admission). No post-operative complications were directly attributable to the gallbladder injury or subsequent cholecystectomy. Patient complications included bacteremia/sepsis (8), acute lung injury (7), intraabdominal abscess in patients with colonic injuries (4), acute kidney injury requiring dialysis (4), wound infection (3), ventilator associated pneumonia (3), deep venous thrombosis (2), abdominal compartment syndrome (1), pulmonary embolus (1), empyema (1), upper gastrointestinal bleeding (1), cerebrovascular accident (1), dehiscence (1), and a pneumothorax related to central line placement (1).

## Discussion

Although the frequency of extrahepatic bile duct injuries in patients with abdominal trauma is moderate (2% to 5%), gallbladder trauma is quite rare [1,2]. Despite seeing well over 40,000 injured patients during the study period at our urban level 1 center, only 45 gallbladder injuries were noted. As observed in other series, the vast majority were caused by a penetrating mechanism [1].

Operative cholecystectomy following injury was initially described in 1887 [6]. Unfortunately it wasn't until 11 years later that the first report of a survivor receiving cholecystectomy after injury was published [6]. By 1905 however, trauma related cholecystectomies were viewed as "brilliant" in both technique and outcome [7]. Despite these successes, the subsequent 60 years witnessed the propagation of suture repair for tears in the gallbladder to ensure organ preservation [8]. Fortunately, this viewpoint has passed with more modern views stating that cholecystectomy again remains the treatment of choice [9]. This remains true regardless of whether injuries are classified as contusions, avulsions, or lacerations. More specifically, theoretical options such as expectant observation, drainage, and cholecystorrhaphy are employed only in the rarest of circumstances [1,2].

The results of our study, the largest series of gallbladder injuries in the literature, supports cholecystectomy as the primary treatment option. Nearly all patients (93%) underwent immediate cholecystectomy. The 3 remaining patients were treated with initial drainage in a rapid fashion prior to application of a temporary abdominal closure technique because the patients displayed physiologic exhaustion secondary to massive hemorrhage from intra-abdominal vascular injuries. Both initial survivors underwent rapid cholecystectomy on their first return to the operating room (24 and 36 hours after presentation respectively). Furthermore, no patients in the series displayed any morbidity or mortality directly associated with the removal of their gallbladder.

As expected, the outcome of patients with gallbladder injuries is synonymous with the mortality induced from their associated injuries [1,2,10-12]. The 6 patients in our series who died within 24 hours were a result of unresuscitatible cardiac, thoracic or intra-abdominal vascular hemorrhage. The remaining 5 delayed patient deaths were caused by multi-organ failure and/or traumatic brain injuries. This pattern is echoed in other series [1,2,13]. Not surprisingly, patients with injuries to the gallbladder present in accordance with the severity of their concurrent trauma. The patients in our series were critically ill with a mean Injury Severity Score of 20 and mortality rate of 24%. Nearly half of our patients also arrived with hemodynamic instability. Each patient in the series displayed an acute abdomen, and therefore an absolute indication for operative exploration. This concept is also supported elsewhere [1,2].

In addition to the observation that all 3 patients with extrahepatic bile duct trauma also possessed injuries to their gallbladder, 10 patients displayed significant trauma to their pancreas. Among this cohort, half underwent simple drainage (grades I and II)[14], while the remainder required a pancreatic resection (4 distal pancreatectomies and 1 delayed pancreatoduodenectomy).

The limitations of this study are multiple. First, it is retrospective and therefore the possibility of bias cannot be eliminated. Second, we were unable to grade gallbladder injuries with the AAST grading system [15] because of poor documentation. Third, we could not evaluate the potential impact of theoretical risk factors for gallbladder injury (thin walled organ [2], high degree of filling [2,6,9,16], and alcohol ingestion [17]), as this information was not captured in patient charts. Finally, although the series is the largest in the literature [1,2,18,19], it is based on a limited number of patients.

## Conclusion

In summary, the safe and rapid treatment for gallbladder trauma is immediate cholecystectomy. The exception to this remains simple drainage in a patient displaying physiologic exhaustion who also receives a temporary abdominal closure and planned operative re-exploration. Injuries are most often caused by penetrating mechanisms, and clinical presentation, as well as patient outcome is a direct result of the severity of their associated injuries.

## Competing interests

The authors declare that they have no competing interests.

## Authors' contributions

CGB, ED and DVF conceived the study, conducted the primary analysis, and drafted the article. AWK, FRS and KBL contributed to the study design and interpretation of all data. All authors contributed to critical revision and approval of the final manuscript.
